# MoS_2_/PPy Nanocomposite as a Transducer for Electrochemical Aptasensor of Ampicillin in River Water

**DOI:** 10.3390/bios11090311

**Published:** 2021-09-01

**Authors:** Maroua Hamami, Meryem Bouaziz, Noureddine Raouafi, Azzedine Bendounan, Hafsa Korri-Youssoufi

**Affiliations:** 1 Université Paris-Saclay, CNRS, Institut de Chimie Moléculaire et des Matériaux d’Orsay (ICMMO), ECBB, Bât 420, 2 Rue du Doyen Georges Poitou, 91400 Orsay, France; maroua.hamami@universite-paris-saclay.fr (M.H.); meryem.bouaziz@synchrotron-soleil.fr (M.B.); 2 Laboratory of Analytical Chemistry and Electrochemistry (LR99ES15), Department of Chemistry, Faculty of Science, University of Tunis El Manar, Rue Béchir Salem Belkheria, Tunis El-Manar, Tunis 2092, Tunisia; noureddine.raouafi@fst.utm.tn; 3 Synchrotron-SOLEIL, Saint-Aubin, BP48, CEDEX, 91192 Gif sur Yvette, France; azzedine.bendounan@synchrotron-soleil.fr

**Keywords:** 2D MoS2, polypyrrole nanoparticles, electrochemical, biosensor, aptamer, antibiotic

## Abstract

We report the design of an electrochemical aptasensor for ampicillin detection, which is an antibiotic widely used in agriculture and considered to be a water contaminant. We studied the transducing potential of nanostructure composed of MoS2 nanosheets and conductive polypyrrole nanoparticles (PPyNPs) cast on a screen-printed electrode. Fine chemistry is developed to build the biosensors entirely based on robust covalent immobilizations of naphthoquinone as a redox marker and the aptamer. The structural and morphological properties of the nanocomposite were studied by SEM, AFM, and FT-IR. High-resolution XPS measurements demonstrated the formation of a binding between the two nanomaterials and energy transfer affording the formation of heterostructure. Cyclic voltammetry and electrochemical impedance spectroscopy were used to analyze their electrocatalytic properties. We demonstrated that the nanocomposite formed with PPyNPs and MoS2 nanosheets has electro-catalytic properties and conductivity leading to a synergetic effect on the electrochemical redox process of the redox marker. Thus, a highly sensitive redox process was obtained that could follow the recognition process between the apatamer and the target. An amperometric variation of the naphthoquinone response was obtained regarding the ampicillin concentration with a limit of detection (LOD) of 10 pg/L (0.28 pM). A high selectivity towards other contaminants was demonstrated with this biosensor and the analysis of real river water samples without any treatment showed good recovery results thanks to the antifouling properties. This biosensor can be considered a promising device for the detection of antibiotics in the environment as a point-of-use system.

## 1. Introduction

Ampicillin (AMP) is a β-lactam antibiotic widely used as a medication for bacterial infections such as pneumonia, bronchitis, and urinary tract infections [1]. It is classified as a broad-spectrum antibiotic, so its use is common in both humans and animals, resulting in the presence of residual amounts in the environment [2]. The frequent use of AMP as an antibiotic has led to the emergence of multi-drug resistant (MDR) bacteria that threaten human health. The presence of MDR bacteria has been found in treated wastewater effluents, with the most common antibiotic resistance being ampicillin (83.3%) [3]. This pollution has numerous impacts on human health, such as epileptic seizures and allergic reactions [4]. Thus, the European Union has therefore set the maximum tolerance limit for the presence of AMPs in certain foods at 50 µg/kg [5]. However, antibiotic residues can be found at higher levels in wastewater or surface waters with concentrations ranging from 1 ng/L to 1 µg/L [6], which is considered a public health problem.

Microbiological testing is generally the method used to determine this class of antibiotics [7], but these tests are not very specific or quantitative, despite their sensitivity. To overcome this limitation, various analytical methods have been used to determine AMP in complex samples, including HPLC [8], mass spectrometry [9], and enhanced surface Raman spectroscopy [10]. Despite the high sensitivity and specificity of these methods, the disadvantage lies in the sample treatment processes which are multi-step, require expensive equipment and must be performed in a laboratory setting. Therefore, a simple, fast, inexpensive, and sensitive system with a portable device allowing the analysis of these contaminants without sample processing is necessary for monitoring river water pollution. Electrochemical biosensors are effective detection tools that meet these criteria and can be used to detect biomolecules as well as organic compounds [11]. Electrode materials play an important role in the analytical properties of biosensors. The nanomaterials used in the design of electrochemical biosensors have a significant effect on the performance of analytical tools. Thus, two-dimensional (2D) nanomaterials have gained attention in the field of electrochemical biosensors due to their electronic properties and high active surface area. The 2D nanomaterial most used in biosensors is graphene. For instance, in the case of antibiotic detection, Wu et al. have developed an electrochemical enzymatic biosensor based on a graphene nanosheet associated with hematite as a pH indicator to detect penicillin residues. The proposed biosensor has a high sensitivity towards the target with a detection limit of 0.1 nM [12]. Similarly, a reduced graphene oxide-based fluorescent aptasensor showed a high sensitivity for the detection of kanamycin. The system was able to detect the antibiotic in various samples such as blood serum and food [13]. Transition metal dichalcogenides (TMDs) have recently received a special attention in biosensor design due to their 2D structures, and their electrocatalytic, optical, and unique electronic properties [14]. The electronic properties of TMD materials are diverse and can be semiconducting, superconducting, or metallic. Molybdenum disulfide (MoS2) belongs to the class of 2D TMD with biocompatible and semiconducting properties. MoS2 has attracted interest due to its thin-film characteristics, which offer a very high surface-to-volume ratio. MoS2 is sensitive to the surrounding environment due to its ultrathin planar structure where electrons/holes are confined in a plane of atomic thickness [15]. Their use in biosensor devices provides high sensitivity. This is related to the sensitivity of the total thickness which is affected when interaction with a target biomolecule occurs. These properties have been explored for FET biosensor design [16] as well as electrochemical biosensors [17,18].

The association of MoS2 with organic materials leads to new properties, such as increased electron transfer capacity, enhanced catalytic activity and stability [19,20]. For instance, the association of MoS2 with reduced graphene oxide (rGO) has been used as a sensor for folic acid by measuring its redox signal [21]. In this biosensor, the similar morphologies of MoS2 and rGO in a layered structure improved their structural compatibility so that they were closely interlinked. These properties improved the electrochemical properties of the folic acid oxidation. The association of MoS2 with conductive polymers such as polyaniline has been also reported by Soni et al. [22] for electrochemical biosensing of cancer biomarkers. The device showed high sensitivity and a wide dynamic range (10–6 M to 10–17 M) for the target DNA with LOD at 3 aM. This sensitivity was explained by the conductive nature of polyaniline and the high active surface area of MoS2.

A MoS2/polypyrrole nanocomposite has also been demonstrated and underlines the enhancement of conductivity and specific surface area [23]. Various electrocatalytic applications in sensing devices have been described for MoS2/PPy composites such as detection of hydrogen peroxide [24] and detection of DNA bases such as guanine and adenine [25]. The MoS2/PPy composite can be obtained by hydrothermal synthesis [26] or by incorporating the MoS2 during polymerization of PPy [27]. Depending on the preparation method of the composite, the properties as well as the structure of the composite can be affected [28]. In the case of the entrapment of MoS2 during formation of the conducting polymers, the monomer fills in the layer of the MoS2 nanosheets leading to strong interactions between Mo ions and nitrogen of pyrrole monomers [23]. The obtained nanocomposite with a sandwich structure has a large surface area, which enhances electrochemical properties [29]. The composite formed by chemical polymerization shows improved conductivity over MoS2 and PPy [30], and has been used as an electrode material for supercapacitors and hydrogen evolution [24]. Exploring the association of MoS2 with nanostructured polypyrrole has not been studied in the literature, to our knowledge. The nanostructured polypyrrole nanoparticles (PPyNPs) should bring a higher surface ratio and improved conductivity. In addition, we explore the formation of such nanocomposite by electrochemical deposition following bottom-up method that could favor electronic interaction between the two nanomaterials and improve the properties of the electrochemical biosensors.

**Scheme 1:**
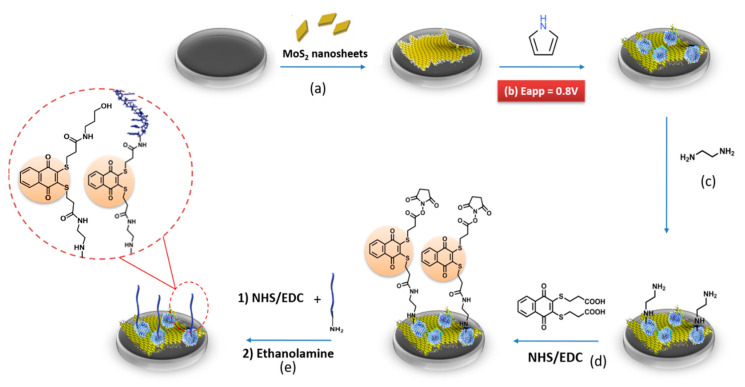
Schematic illustration of the various steps for the design of MoS2/PPy based biosensor, (**a**) MoS2 nanosheets electrodeposition, (**b**) PPyNPs obtained by pulsed electropolymerization, (**c**) EDA electrodeposition, (**d**) redox marker covalent attachment and (**e**) aptamer immobilization.

In this work, we develop an aptasensor for ampicillin detection based on a nanocomposite comprising MoS2/PPyNPs formed with the electrodeposition method using a layer-by-layer strategy. The ampicillin-binding aptamer was covalently attached to the surface of the nanocomposite. Naphthoquinone (NQ) used as a redox marker was immobilized on the modified electrode surface (Scheme 1). The sensing device explores the electrocatalytic effect of the MoS2 and PPyNPs as a conducting surface to improve the electronic and structural properties of nanocomposite. The biosensor platform was obtained by rationalized deposition method, which improves the electronic properties of the nanocomposite and the redox properties of NQ. In addition, the high surface ratio provided by nanostructure of the nanocomposite should improve the loading of the aptamer on the surface.

## 2. Material and Methods

### 2.1. Reagents

Hexaammonium heptamolybdate tetrahydrate, thiourea, 1,4-naphthoquinone, 3-mercaptopropionic acid, N-(3-dimethylaminopropyl)-N-ethylcarbodiimide hydrochloride (EDC), pyrrole, lithium perchlorate, ethylendiamine (EDA), cystamine, ethanolamine, phosphate-buffered saline (PBS), ampicillin, levofloxacin, amoxicillin, and penicillin were purchased from Sigma-Aldrich (France). All the PBS solutions (pH = 7.4) were prepared by dissolving one tablet in 200 mL (0.01 M) then filtered using a 0.22 μm membrane filter and stored at 4 °C until use. The aptamer was provided by Eurogentec company. The used sequence is: 5′-NH2-(CH2)6-GCGGGCGGTTGTATAGCGG-3′ with Kd = 13.4 nM [31].

Other chemicals used in this work were of analytical grade and directly used without additional purification. All solutions were prepared with Milli-Q water (18 MΩ cm^−1^) from a Millipore system. Real sample assays for environmental water sample control were collected from the Majerda river (Beja) in Tunisia.

### 2.2. Methods

Atomic force microscopy (AFM) images were obtained with tapping mode topography and phase imaging using an Innova AFM Bruker with NanoDrivev8.02 software. Tapping mode images were acquired using silicon tips from Nanosensors (PPP NCSTR) with a resonance frequency ranging between 76 and 263 kHz. Scanning electron microscope (SEM) micrographs and energy dispersive X-ray (EDX) spectra were recorded using a FEI Quanta 200 Environmental SEM.

1H and 13C NMR spectra were recorded on a Bruker Advance 300 MHz apparatus. The chemical shifts are given in ppm according to dimethyl sulfoxide-d6 (d6-DMSO) using tetramethylsilane as an internal reference. Raman spectra were obtained at room temperature by a Raman spectrophotometer from Horiba Jobin Yvon with a CCD detector. FT-IR characterizations were obtained using a Bruker IFS66 FT-IR spectrometer (Bruker, Germany) equipped with a mercury cadmium-telluride (MCT) detector and an attenuated total reflectance (ATR) germanium crystal.

The high-resolution photoemission experiments were carried out using the synchrotron radiation at TEMPO beamline of Synchrotron SOLEIL, France. The XPS data were recorded by means of an MBS A^−1^ analyzer. The measurements were made with pass energy of 100 eV and the photon energy used for the core levels shown here is 580 eV. The overall energy resolution (beamline and spectrometer) is estimated to be less than 0.1 eV. All the XPS spectra were calibrated with Au 4f7/2 at a binding energy of 84 eV on a clean Au substrate.

The electrochemical measurements: cyclic voltammetry (CV) and electrochemical impedance spectroscopy (EIS) were recorded using a Metrohm Autolab PGstat 12 controlled by Nova^®^ software and square wave voltammetry (SWV) was recorded using μStat8000 Multi Potentiostat/Galvanostat controlled by DropView 8400. The screen-printed electrodes used are from DropSens reference DRP-C11L formed with a carbon electrode (4mm diameter) as the working electrode, auxiliary electrode of carbon and reference electrode of Ag/AgCl.

### 2.3. Synthesis of the Naphthoquinone Derivative

The synthesis of modified redox marker was performed following the method described in [32]. 3-mercaptopropionic acid (0.90 mL, 10.0 mmol) was added to a solution of (1,4-naphthoquinone-2-yl)-mercaptopropionic acid (0.80 g, 3.0 mmol) in 20 mL of ethanol. Then the mixture was heated at reflux for 2 h and the reaction was monitored by TLC (silica/EtOAc). The solvent was removed under reduced pressure to obtain a brown solid. The obtained product was added to a mixture of 20 mL of ethanol and 100 mL of hexane and left to stir overnight. The reaction mixture was filtered to yield 3,3′-(1,4-dihydro-1,4-dioxo-2,3-naphthylenedithio)dipropionic acid (0.48 g, 42.8%) as a yellow powder.

1H NMR (d6-DMSO) δ: 2.65–2.69 (t, 2H), 3.11–3.12 (t, 2H), 6.77 (s, 1H), 7.81–7.86 (m, 2H), 7.95–7.97 (m, 2H); 13C NMR (d6-DMSO) δ: 24.76, 31.95, 126.03, 126.34, 127.28, 131.43, 131.68, 133.65, 134.74, 153.40, 172.48, 180.95, 181.83.

### 2.4. Preparation of MoS2 Nanosheets

The ultrathin MoS2 nanosheets were prepared by a one-step hydrothermal method following the same procedure as reported by Zhang et al. [33]. The reagents, 1.24 g of hexaammonium heptamolybdate tetrahydrate and 2.28 g of thiourea were dissolved in 36 mL of deionized water and left under vigorous stirring for 30 min to form a homogeneous solution. Then, the solution was transferred into a tightly sealed 50 mL Teflon-lined stainless-steel autoclave and heated to 220 °C. After 24 h the mixture was allowed to cool down to RT. After the centrifugation, the black precipitate was collected and washed with distilled water and pure ethanol several times. Finally, the product was dried in vacuum at 60 °C for 24 h. The obtained MoS2 nanomaterial was characterized by Raman spectroscopy (Figure S1A) and EDX (Figure S1B).

### 2.5. Modification of SPCE with MoS2 or PPy

The deposition of MoS2 and PPy was performed by electrochemical deposition on the surface of screen-printed carbon electrode (SPCE). Briefly, 5 mg/mL of MoS2 nanosheets were dispersed under sonication in 0.5 M LiClO4 aqueous solution. The SPCE was covered with 50 µL of the prepared solution and the potential was swept from 0.20 V to −0.95 V vs. Ag/AgCl with scan rate of 50 mV s^−1^ for 10 cycles (see, SI, Figure S2). Then, the modified electrode was rinsed several times with deionized water and dried under a gentle flux of N2.

The immobilization of polypyrrole film on SPCE and SPCE/MoS2 was performed with the same method after optimization. A solution of 50 µL containing 10 mM of pyrrole in a 0.5 M LiClO4 solution was used to cover the SPCE electrode, then potential was swept within two pulses from 0 to 0.8 V over 5 s (see, SI, Figure S3A). The number of pulses was optimized to obtain thin layer of nanostructured polypyrrole on SPCE and SPCE/MoS2 surfaces (See SI, Figure S3B).

### 2.6. Formation of Ampicillin Aptasensor

Surface functionalization by the amino groups was achieved by the electrodeposition of EDA on the MoS2/PPy nanocomposite using CV through amine oxidation following the same approach described previously [34]. EDA (6 mM) was prepared in an aqueous 0.5 M LiClO4 solution and deposited on the electrode surface, the potential was then scanned between −0.2 and 0.8 V for two cycles at a scan rate of 200 mV s^−1^ to covalently link EDA on the polypyrrole layer and to obtain the aminated surface (see SI, Figure S4A).

The redox marker, naphtoquinone-terminated dithiopropanoic acid was covalently attached to the terminal amines present on EDA/PPy/MoS2 and EDA/PPy by incubating the electrode in a solution containing 10 mM of EDC and catalytic amount of NHS for 1 h following the optimized method [35]. The electrode surface was thoroughly washed with PBS to remove non-attached NQ molecules. Finally, incubation of electrodes in 10 μM of the ampicillin-binding aptamer functionalized with an amino group in the 5′ position, for 30 min at 35 °C, lead to their covalent attachment through an amide bond.

To prevent the non-specific interaction by adsorption of non-specific target a blocking step was achieved by covering the surface of the electrode for 30 min with 1 mM ethanolamine. The biosensor was finally stored overnight in PBS solution at 4 °C for stabilization.

### 2.7. Aptamer Binding to Ampicillin

The aptamer used in this study presents a high specificity to ampicillin and offers high stability over the time [36]. The target solutions were prepared at different concentrations by diluting a stock solution with PBS. The target detection was achieved at RT by incubating the biosensor for 30 min in 50 µL of different concentrations of AMP, followed by a washing step with buffer solution.

## 3. Results and Discussion

### 3.1. Nanocomposite Formation

The nanocomposite MoS2/PPy modified electrode was prepared using electrodeposition technique (Scheme 1). Firstly, a MoS2 dispersion was deposited on the SPCE by electrochemical process. This method ensures high attachment of the 2D nanomaterials on the carbon surface through hydrophobic interaction [37]. Weak interlayer Van der Waals interactions allow MoS2 to act as an efficient host for a variety of electron donating atoms on the SPCE surface [38]. This “nano to nano” approach presents many advantages compared to the conventional electrodeposition methods since it can be carried out under mild conditions: at moderate potential, at RT, and in aqueous solutions, and the obtained coating keeps the properties of the deposited nanomaterials. According to Liu et al. [39] this “redox free” process applied to form nanomaterial coatings from their aqueous dispersions without involving oxidation–reduction enables retention of the nanomaterials’ intrinsic properties. This approach is based on applying an electrical potential decreasing the inter-particle repulsive forces that ensure the nanomaterials dispersion stabilization. It has been reported, in most cases, that the oxidation or reduction of water occurs upon application of electrical potential which will lead to a pH modification on the electrode surface and suppression of the net surface charge of the dispersed nanomaterials, causing their aggregation and irreversible deposition on the electrode surface [40].

The SPCE/MoS2 electrode was then modified with polypyrrole through electrochemical polymerization with pulsed potential sweep. This approach is based on two stages for PPy nanoparticles formation: an ON time period where a constant potential was applied corresponding to the oxidation of pyrrole and an OFF time period where the system is at rest. During the ON time pulse period, the pyrrole monomers in the solution were oxidized to radical cations and subsequently coupled to form PPy nanoparticles, which were deposited on the surface of the MoS2. In the following OFF time pulse, the growth of PPy nanoparticles was obstructed or, when the OFF time was relatively long, the already-grown chains could undergo oriented growth over the surface [41]. During the next pulse, nucleation and growth of PPyNPs will occur on new sites of the surface. As such, polypyrrole nanoparticles with controllable structure could be formed. This method favors the growth of polypyrrole in the various nucleation sites, with the electropolymerization of pyrrole monomers rather than nucleation and growth on existing polymer chains overcoming the cauliflower surface morphology [41] and favoring the PPy nanoparticle structure.

### 3.2. Chemical and Electronic Properties of MoS2/PPy Nanocomposite

**Figure 1:**
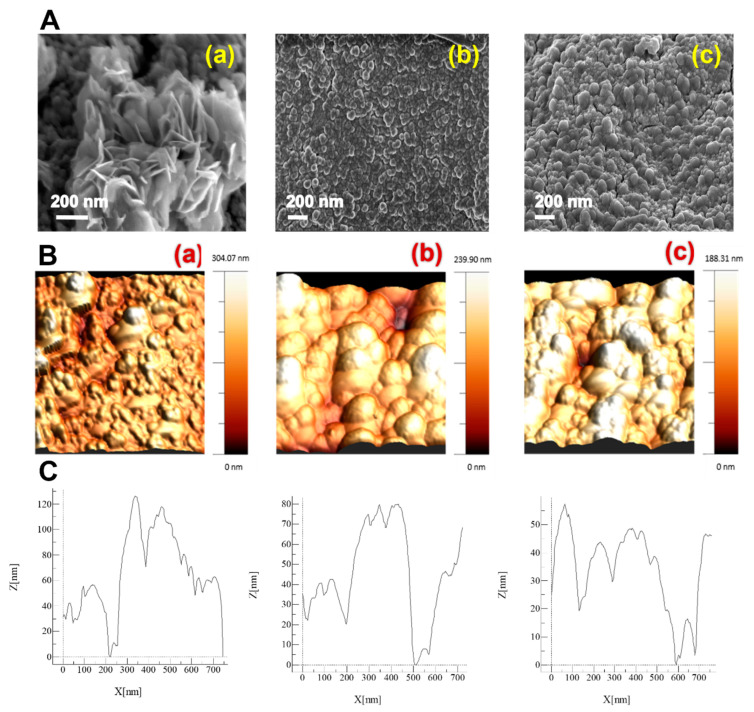
(**A**) SEM images, (**B**) AFM images and (**C**) rugosity profiles of various SPCE modified electrodes: (**a**) MoS2 nanosheets; (**b**) PPyNPs obtained by pulsed electropolymerization, and (**c**) MoS2 /PPyNPs nanocomposite.

The surface morphology analysis of nanocomposites was examined with SEM and AFM. SEM observations were performed on various modified SPCEs (Figure 1) to confirm the nano-structure formation. Electrodeposition of MoS2 nanosheets leads to multilayered nanosheets and aggregates with a vertical orientation. This could be explained by the interaction between different MoS2 islands during electrodeposition (Figure 1A, image a). The electrode modified with PPyNPs obtained via pulsed electropolymerization showed the formation of the polymer nanoparticles with a controllable structure and oriented growth over the surface (Figure 1A, image b). This is different from electropolymerization of pyrrole with cyclic voltammetry, which typically exhibits cauliflower-like morphology with irregular growth of polypyrrole film over the surface (see SI, Figure S5B). The SEM image of SPCE/MoS2/PPyNPs shows nanoparticles covering the MoS2 nanosheets and presenting a homogenous distribution over the surface (Figure 1A, image c).

AFM studies were obtained as a complementary information. MoS2 surface imaging shows homogenous nanoaggregates with a high roughness factor (Figure 1B,C, curve a). Upon modification with PPyNPs, a change in the surface morphology with less aggregation in the nanoparticle structure was observed (Figure 1B, image c) with lower roughness. The shape is the same as the modified SPE with PPyNPs (Figure 1B, image b), which demonstrated that PPyNPs cover the surface of MoS2 and provide a large surface ratio.

The elemental analysis of the compositions of the MoS2/PPyNPs was performed using energy dispersive X-ray EDX. The spectra obtained confirmed the presence of molybdenum and sulfur in both modified surface SPCE/MoS2 and SPCE/MoS2/PPyNPs (See SI, Figure S6A). In the case of MoS2/PPyNPs, EDX showed the presence of C, N related to polypyrrole, Mo, S related to the MoS2 and the Cl, O due to the presence of ClO4− used in the electrochemical polymerization process and remaining as doping ions.

The structural properties were also analyzed through FT-IR analysis. Thus, MoS2, PPyNPs and MoS2/PPyNPs were characterized after electrochemical deposition on SPCE using the attenuated total reflectance method. The obvious characteristic peaks of polypyrrole at 775, 1160, 1292, 1448, and 1551 cm^−1^ were observed in both PPyNPs and MoS2/PPyNPs (see SI, Figure S6B) with some variation. The peak located at 1160 cm^−1^, corresponding to the stretching vibration of C-N bond, remains at the same wavelength in the nanocomposite. However, the peak located at 1551 cm^−1^ corresponding to the vibration of pyrrole ring shifts to 1525 cm^−1^ in the case of MoS2/PPyNPs. This behavior is related to the interaction of PPyNPs with MoS2 [42].

**Figure 2:**
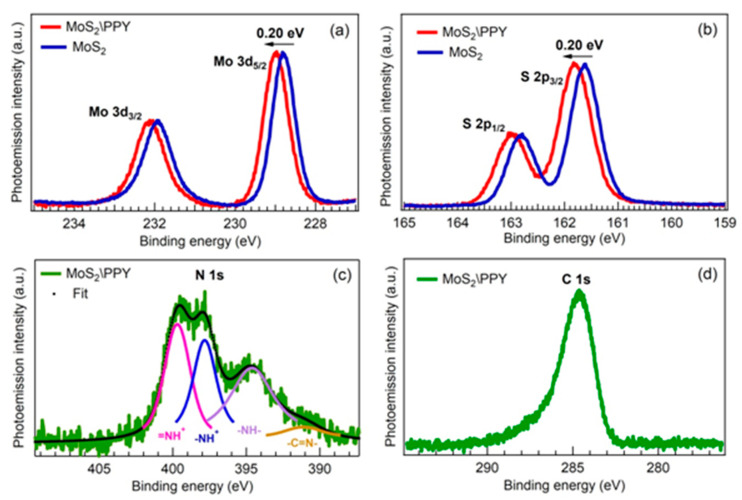
High-resolution XPS core-level spectra of (**a**) Mo3d, (**b**) S2p, (**c**) N1s, and (**d**) C1s measured on MoS2 nanosheets and on MoS2/PPyNPs.

High-resolution XPS measurements were performed to study the electronic properties of the MoS2 nanosheets and the MoS2/PPyNPs nanocomposite. Different core levels were probed, namely Mo3d, S2p, N1s, and C1s. As shown in Figure 2a, the Mo3d spectrum measured on MoS2 shows two distinct peaks at 231.9 eV and 228.8 eV, which are respectively associated with the Mo4+3d3/2 and Mo4+3d5/2 spin-orbit components of the semiconducting 2H phase, which is in agreement with the literature [26]. Also, the S2p spectrum displays two spectroscopic features at 162.81 eV and 161.63 eV corresponding to S2p1/2 and S2p3/2, respectively [28]. For the MoS2/PPyNPs, we observe a shift of about 0.20 eV to a higher binding energy for both the Mo3d and S2p core levels. Such a shift is related to the interaction of the polypyrrole backbone with the MoS2 nanosheets and represents proof of a charge transfer process between the two nanomaterials. The energy transfer led to the formation of a new nanomaterial with an improved conductivity.

The deconvolution of the N1s XPS spectrum recorded on the MoS2/PPyNPs nanocomposite displayed different spectroscopic components, as indicated in Figure 2c. For instance, the N1s peak deconvolution showed four components: at 399.70, 397.84, 394.60, and 391.12 eV, which could be attributed to (–N–H•+), (=N–H+), (–N–H), and (=N–), respectively [43]. In line with the literature, the positively charged nitrogen of polaron (–N–H•+) and bipolaron (=N–H+) species are related to the higher binding energies whereas the imine (=N–) structure corresponds to the lowest binding energy at 391 eV. The component at ~394.60 eV is attributed to neutral nitrogen in the pyrrole ring (–N–H structures). All the components showed a shift toward lower energy compared to PPy where the binding energy values were obtained at 402.58, 401, 399.85, and 398.88 eV [44]. This large energy shift demonstrates a strong modification of the PPyNPs properties due to their interaction with MoS2. The C1s spectrum presented in Figure 2d allows us to exclude any undesirable damage to the molecules.

### 3.3. Electrochemical Properties of MoS2/PPyNPs

The electrochemical properties of MoS2, PPyNPs, and MoS2/PPyNPs modified electrodes were investigated in phosphate buffer saline containing 5 mM of [Fe(CN)6]^3−/4−^ using cyclic voltammetry and EIS to underline the electron transfer ability and the electrical properties of the nanomaterials.

**Figure 3:**
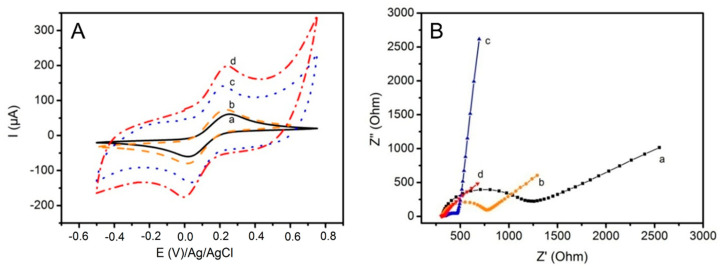
Electrochemical characterization in solution of 5 mM [Fe(CN)6]^3/4−^, 0.1 M KCl solution. (**A**) CV obtained with scan rate 100 mV/s and (**B**) EIS obtained with frequency range: 100 KHz to 0.1 Hz (**a**) SPCE, (**b**) SPCE/MoS2, (**c**) SPCE/PPyNPs, and (**d**) SPCE/MoS2/PPyNPs.

The CV of MoS2 shows a slight increase in the peak current value of 7.36 µA (Figure 3A; curve b) in comparison to the SPE carbon electrode (Figure 3, curve a). In the case of the PPyNPs nanostructure, an important increase was observed with a double value (14.14 µA) (Figure 3A, curve b). MoS2/PPyNPs modified electrodes showed an increase in current to 19.83 µA (Figure 3A, curve d). This improvement in redox current is attributed to the hybrid structure of MoS2/PPyNPs with an efficient electron transfer due to a synergetic effect of the catalytic properties of MoS2, the large surface area provided by the nanostructure, and the conductivity of the resulting layer.

The characterization of modified electrodes was also performed with EIS to analyze the electrical interfacial properties of the various nanomaterials (Figure 3B). A Nyquist plot showed the variation of semicircle diameter depending on the modified surface which is related to charge transfer resistance (Rct). The fitting data using the Randell equivalent circuit model shows the electrical parameters of the various modified electrodes MoS2, PPyNPs, and MoS2/PPyNPs (see Table S1).

Modification of the SPCE with MoS2 induced a decrease in the Rct from 849 Ω to 412 Ω, demonstrating enhancement of charge transfer ability (Figure 3B, curve b). The Rct of PPyNPs reached 197Ω (Figure 3B, curve c). When conducting PPyNPs were formed on the surface of MoS2, a large drop in the Rct value was measured, reaching 20 Ω (Figure 3B, curve d). Therefore, modification with the nanocomposite MoS2/PPyNPs lead to a decrease in the Rct value, showing an improvement in the conductivity and confirming the synergic effect demonstrated by the XPS data.

### 3.4. Biolayer Formation and Characterization

The introduction of functional groups on the surface was performed by modification with EDA following an electrochemical approach through amine oxidation. The EDA could be linked to polypyrrole by covalent bonding formed by the electro-oxidation of the terminal amines that yield radicals which react with the polypyrrole NPs [34]. In the case of MoS2 based platform, cystamine was used instead of EDA due to the high affinity of molybdenum to sulfur [45].

The amino groups displayed on the surface provided by the EDA film served to the covalent attachment of the redox markers. The immobilization of NQ bearing carboxylic acid groups in the terminal position was achieved in the presence of EDC as a coupling agent and the second function allowed the further attachment of the aptamer through amid bonds. The stepwise modification of the electrodes was monitored by CV using [Fe(CN)6]^3/4−^ as redox probe (see SI, Figure S7).

**Figure 4:**
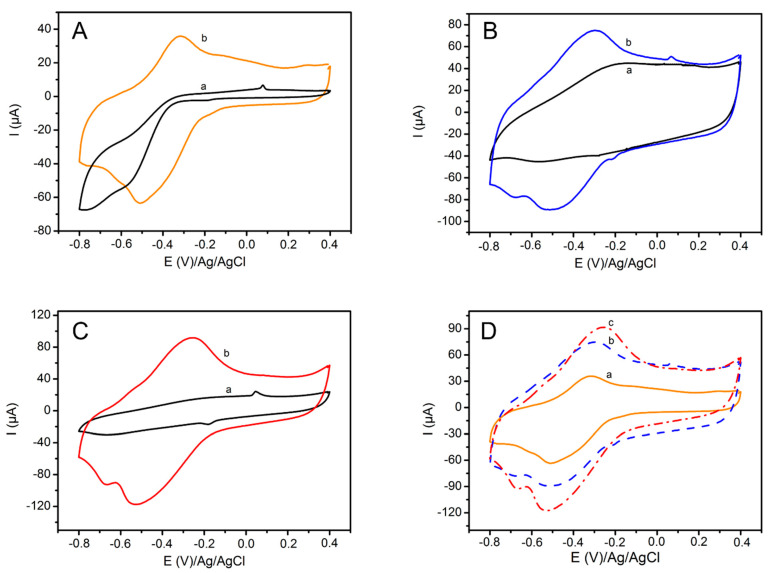
CVs recorded in 10 mM PBS pH 7.4 at scan rate 100 mV/s: (**A**) MoS2, (**B**)/PPyNPs, (**C**) MoS2/PPyNPs; (**a**) before and (**b**) after NQ attachment); (**D**) CV comparing the three platforms after NQ attachment: (**a**) MoS2, (**b**) PPyNPs, and (**c**) MoS2/PPyNPs.

The immobilization of the redox marker NQ was also followed by CV in PBS solution free of redox marker and by following its redox signal. The CVs of modified MoS2/NQ, PPyNPs/NQ and MoS2/PPyNPs/NQ show a redox signal after NQ attachment for all modified SPEC surfaces (Figure 4). The variation between anodic and cathodic peaks recorded for all the platforms was greater than 59/n mV, which demonstrates a quasi-reversible system of NQ. The CV of the MoS2/PPyNPs/NQ (Figure 4D, curve c) shows a shift in the redox signal towards negative potential compared to the CV of MoS2 (Figure 4D, curve a) and PPyNPs (Figure 4D, curve b) and higher redox current. This result suggests that the nanocomposite exhibits good electrocatalytic activity towards NQ reduction.

The effect of varying the scan rate on the response of the attached NQ was also investigated for all the platforms (see SI, Figure S8A,C,E). In all cases, both peak currents increased with the scan rates with a slight shift of the anodic peak and cathodic peak potential. The anodic and cathodic peak currents were linearly correlated to scan rate in the range from 50 to 500 mV·s^−1^ (see SI, Figure S8B,D,F). This result is representative of an adsorption-controlled electrode process.

The immobilization of the aptamer was also followed by CV and EIS in the presence of [Fe(CN)6]^3/4−^ (see SI, Figure S9) and showed a decrease in current in the case of CV. An increase in the Rct in the case of EIS studies is in agreement with the successful covalent attachment of negatively charged aptamers on the surface of the biosensor blocking electron transfer.

### 3.5. Comparison of the Sensing Platforms Based on MoS2

**Figure 5:**
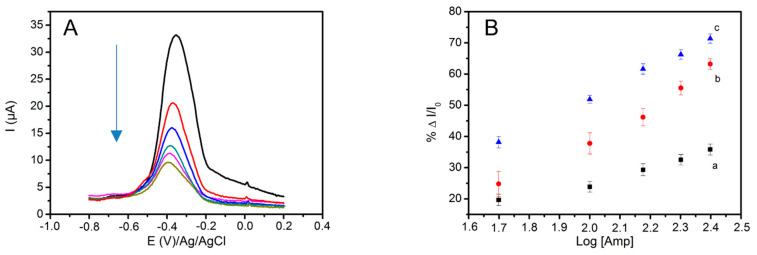
(**A**) SWVs of the oxidation peak after incubation at ampicillin concentrations of 50, 100, 150, 200, and 250 pg/L in PBS for the MoS2/PPy based electrode; (**B**) calibration curve of the biosensor displaying the relative changes of the current peak % ∆I/I0vs Log [AMP] for (**a**) SPCE/MoS2/NQ/APT, (**b**) SPCE/PPyNPs/NQ/APT, and (**c**) SPCE/MoS2/PPyNPs/NQ/APT.

The sensing activity of the developed biosensors towards AMP was evaluated using SWV. Therefore, the modified electrode was incubated in solutions at different concentrations of AMP leading to a decrease in the NQ redox current (Figure 5A). This current decrease can be explained by the formation of the aptamer-target complex, which hinders the electron transfer and the proton diffusion required to protonate the reduced form of the naphthoquinone. The resulting current variation was proportional to the target concentration. A control experiment was run to compare the biosensor response with different electrode materials, MoS2 and PPyNPs, to underline the added value of the hybrid MoS2/PPyNPs association in ampicillin detection. The measurements were performed at the same experimental conditions for MoS2 and PPyNPs (see SI, Figure S10) and demonstrated a decrease in the redox signal of NQ after ampicillin detection. Calibration curves presenting the average variation in current after the successive addition of AMP with a logarithmic scale of concentration were plotted (Figure 5B). The variation was linearly proportional to the log of the AMP concentration for the three biosensors. A linear regression equation of %∆I/I0 = −42.73 + 47.57 log[target]/(pg/L) (R = 0.998) (Figure 5B, curve c) was obtained for the MoS2/PPyNPs biosensor, with ∆I/I0 = −35.34 + 29.62log[target]/(pg/L) (R = 0.997) and % ∆I/I0 = −91.11 + 63.91 log[target]/(pg/L) (R = 0.977) were found for MoS2 and PPyNPs biosensors, respectively.

The relative standard deviation (RSD) was calculated for four fresh biosensors. The value obtained was 1.78% for the MoS2 platform which owes its reproducibility in aqueous solution to the electrochemical deposition methods and the stability of the layers. In the case of biosensor formed with PPyNPs, an RSD of 2.83% was obtained. The main drawback of the PPy film is its ability to undergo irreversible degradation (or over-oxidation by exposure to ambient conditions). The over-oxidized polymers lose their properties, for example, conductivity and redox activity. In the case of the nanocomposite MoS2/PPyNPs, an RSD of 1.54% was obtained which demonstrates that combining these two materials confers to the biosensor good reproducibility and stability.

The LODs calculated based on the (3S/N) method were 10 pg/L for MoS2/PPyNPs and increased to 66 pg/L and 71 pg/L for MoS2 and PPyNPs, respectively. The detection domain studied in this work does not include the concentration values of the maximum levels of ampicillin set by the European Union. It is worthy to note that studies showed that the degradation of ampicillin is almost complete and reaches a maximum after 48 h in rivers [46]. Therefore, taking into consideration this degradation step, it is of high interest to develop an aptasensor for ampicillin detection in samples with very low antibiotic concentrations.

The properties of the present sensors are improved compared to other electrochemical biosensors using other nanomaterials (Table 1) and the same order of sensitivity can be obtained with advanced techniques such as photoelectrochemical methods. However, in this biosensor various advantages are obtained besides the high sensitivitthe fabrication process by patterning through electrodeposition of MoS2 and PPyNPs on SPCEs as well as the covalent attachment which leads to high stability and reproducibility of the biosensors. This electrochemical patterning approach opens the way to the integration of these biosensors in multi-detection systems. Electrochemical detection through amperometric measurement is also an advantage regarding the electrochemical instrumentation and the possible use of handheld devices.

### 3.6. Analytical Performance

**Figure 6:**
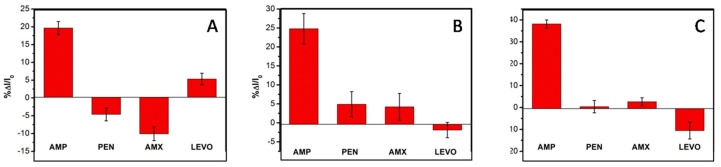
Histograms giving the current percentage after incubation in different interferents for (**A**) MoS2 based biosensor, (**B**) PPy based biosensor, and (**C**) MoS2/PPyNPs based biosensor.

Evaluation of the biosensors’ selectivity was also carried out by studying the response of the different platforms in presence of others antibiotic such as penicillin (PEN), amoxicillin (AMX), and levofloxacin (LEVO). We compared the biosensor’s response with responses obtained when incubated in excess amounts of the aforementioned substrates (concentration 10 times higher than that of AMP (50 ng. L^−1^). The results obtained from SWV are represented in histograms (Figure 6). The latter indicates that MoS2 exhibits a good selectivity towards penicillin and amoxicillin (Figure 6A) while PPyNPs platform was more selective to levofloxacin (Figure 6B). However, the hybrid MoS2/PPyNP-based biosensors showed high selectivity to all interferents (Figure 6C), demonstrating the effect of the nanomaterials and biosensor fabrication approach for selectivity enhancement. However, the presence of levofloxacin shows a negative response which could be related to its redox properties that could promote electron transfer of the hole system [50].

**Figure 7:**
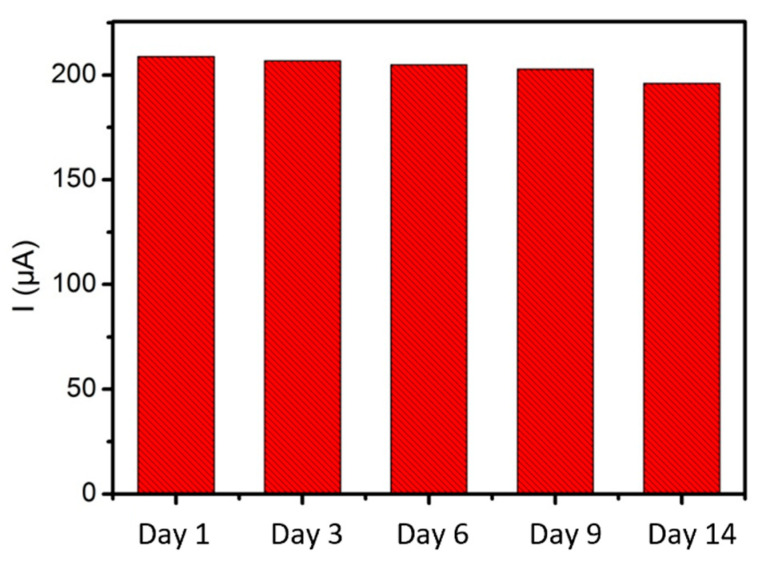
Histogram of the storage stability test.

The stability was also checked by analyzing the electrochemical behavior of the formed nanocomposite MoS2/PPyNPs in a solution containing redox marker [Fe(CN)6]^3/4−^ over two weeks. The obtained results are represented in a histogram (Figure 7), showing that the electrochemical response remained the same and the nanocomposite maintained its electrochemical properties, which underlines the high stability of the nanocomposites enhanced by the presence of MoS2.

### 3.7. Detection in River Water

**Figure 8:**
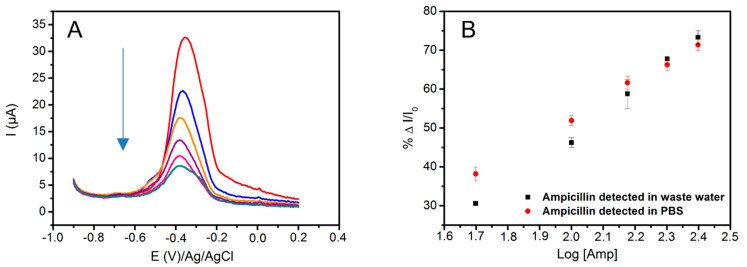
(**A**) SWV showing the oxidation peak of the biosensor after the incubation at various ampicillin concentrations 50,100, 150, 200, and 250 pg/L in river water. (**B**) Calibration curve of the biosensor displaying the relative changes in the current peak % ∆I/I0 vs. Log [AMP] for ampicillin detection in river water (Black) and in PBS (Red).

Real-world application of the developed biosensor was demonstrated in water samples collected from the most polluted river in Tunisia (Beja). The samples were first concentrated and then analyzed by HPLC and mass spectroscopy to control for the presence or absence of ampicillin. The sample showed no sign of ampicillin with these methods (see SI, Figure S11). The sample was then spiked with a standard solution of the AMP to obtain final a concentration from 50 to 250 pg/L. The biosensor was then incubated in the prepared solutions (Figure 8A). The measurement was repeated with the three different biosensors. The results obtained from the sample of AMP in river water were compared to the values obtained in PBS solution to underline the matrix effect. First, the calibration curve obtained from river water was plotted and compared to that obtained in PBS. The curve shows a linear variation with the log of concentration of AMP with regression equation of %∆I/I0 = −74.86 + 61.95 log[target] (pg/L) (R = 0.998) (Figure 8B). The slope is bit higher than that obtained in the PBS solution and highlights a low matrix effect related to the composition and the components of the river water. The obtained results underline the potential of the developed biosensor based on the MoS2/PPyNPs nanocomposite for the river water pollution monitoring with SPCE and handheld electrochemical devices.

## 4. Conclusions

In this study, MoS2 nanosheets were successfully associated to PPyNPs through a layer-by-layer strategy using electrodeposition methods. The obtained MoS2/PPyNPs composite was applied as an efficient and stable electrode material for the design of an ampicillin aptasensor. We highlighted improvements in the structural and electronic properties of MoS2/PPyNPs obtained by this approach and demonstrated by high-resolution XPS a core level energy shift and charge transfer process between the two nanomaterials. The energy transfer led to the formation of new nanomaterials with improved conductivity of the entire system. In addition, the nanocomposite MoS2/PPyNPs offers a large electroactive surface area and electrocatalytic activity. These properties contributed to the enhancement of their electrochemical performance towards attached naphthoquinone used as a redox marker. The hybrid MoS2/PPyNPs modified with aptamer showed enhanced analytical performance and high selectivity towards ampicillin compared to MoS2 and PPyNPs.

The study of ampicillin in water collected from a river showed a low matrix effect. The developed biosensor is a promising tool for application in hand-held devices for monitoring organic pollutants in rivers using tailored aptamers.

## Figures and Tables

**Table 1:** Properties of various biosensors developed for ampicillin.

Platform	Detection Method	LOD	Ref
GCE/AuNPs	DPV	32 pM	[47]
PEDOT:TsO/PEDOT-OH:TsO bilayer	EIS	100 pM	[48]
ITO/BiFeO_3_/utg-C_3_N_4_	Photoelectrochemical	0.33 pM	[4]
ITO/ZnxCo_3−x_O_4_/N-GQDs/AgBiS_2_	Photoelectrochemical	0.25 pM	[49]
SPCE/MoS_2_/PPyNPs/NQ	SQW	0.28 pM	This work

GCE: glassy carbon electrode, AuNPs: gold nanoparticles, PEDOT: TsO; tosylate-doped poly(3,4-ethylenedioxythiophene), PEDOT-OH:TsO: hydroxymethyl derivative, ITO: indium-tin-oxide, p-type BiFeO_3_ nanoparticles, utg-C_3_N_4_: n-type carbon nitride nanosheets, N-GQDs: nitrogen-doped graphene quantum dots, ZnxCo_3−x_O_4_: bimetallic oxide.

## Data Availability

Not applicable.

## Supplementary Materials

The following are available online at https://www.mdpi.com/article/10.3390/bios11090311/s1, Figure S1: (A) Raman Spectra of MoS2 nanosheets and (B) EDX analysis of the modified SPCE with MoS2 nanosheets; Figure S2: Electrodeposition of MoS2by cycling the potential between −0.1 V and −0.95 V, for 10 cycles at scan rate 100 mV·s^−1^ in 0.5 M LiClO4; Figure S3: (A) Electropolymerization of PPy by 2 pulses with Eapp = 0.8 V during 5 s in 0.5 M LiClO4, and (B) CVs recorded in 5 mM [Fe(CN)6]^3/4−^ at v = 100 mV·s^−1^ before and after PPy elecropolymerization (a) SPCE (b) 1 pulse (c) 2 pulses (d) 3 pluses and (e) 4 pulses, Figure S4: (A)CVs of Electrodeposition of EDA by cycling the potential between −0.2 V and 0.8 V, for 2cycles at scan rate 200 mV·s^−1^, in 0.5 M LiClO4, (B) Electrodeposition of Cystamine by cycling the potential between −0.2 V and 1.2 V, for 4 cycles at scan rate 100 mV/s, in 0.5 M LiClO4; Figure S5: SEM images of (A) SPCE (B) SPCE/PPy Polypyrrole deposits using cyclic voltammetry; Figure S6: (A) EDX analysis of the modified SPCE with MoS2/PPy and (B) FT-IR MoS2 nanosheets, PPy and MoS2/NPsPPy, Figure S8: CVs of the redox signal of NQ in various modified SPCE in PBS pH= 7.4 with scan rate from 50 to 500 mV·s^−1^ and Linear relationship of the redox peak currents versus scan rate (A,B) MoS2/Cyst/NQ, (C,D) PPy/EDA/NQ, (E,F) MoS2/PPy/EDA/NQ; Figure S9: CVs recorded in presence of 5 mM [Fe(CN)6]^3/4−^ before and after aptamer immoabilization (A.a) SPCE/MoS2/PPy/EDA/NQ; (A.b) SPCE/MoS2/PPy/EDA/NQ/APT; (B.a) SPCE/MoS2/Cyst/NQ, (B.b) SPCE/MoS2/Cyst/NQ /APT; (C.a) SPCE/PPy/EDA/NQ; (C.b) SPCE/PPy/EDA/NQ/APT; EIS (Nyquist plots) in presence of 5 mM [Fe(CN)6]3/4– obtained before (D) (i) and after (D) (ii) aptamer immobilization (Eapp = 0.2V ) Frequency range: 100 KHz to 0.1 Hz; Figure S10: SWV of the oxidation peak after incubation at various ampicillin concentrations from 50 to 250 pg/L in PBS for (A) MoS2 based electrode (B) PPy based electrode; Figure S11: Mass Spectra of (a) water doped with AMP and (b) river water (B) High performance liquid chromatography (HPLC) analysis of (a) Ampicillin solution and (b) waste water sample; Table S1: Fitting parameters of EIS data.
