# Supplementary material for: MoS2/PPy Nanocomposite as a Transducer for Electrochemical Aptasensor of Ampicillin in River Water
Source: Biosensors (Basel). 2021 Sep 1;11(9):311. doi: 10.3390/bios11090311 (PMC8466510; doi:10.3390/bios11090311)
Supplement: Supplementary file 1 [file biosensors-11-00311-s001.zip › biosensors-1342342-supplementary.pdf]

SUPPLEMENTARY MATERIAL

MoS_2/PPy nanocomposite as transducer for electrochemical aptasensor of ampicillin in river water

M. Hamami[a b], M. Bouaziz[a c], N. Raouafi[b], A. Bendounan[c], H. Korri-Youssoufi[a]*

[a]Université Paris-Saclay, CNRS, Institut de Chimie Moléculaire et des Matériaux d'Orsay (ICMMO), ECBB, Bât 420, 2 Rue du Doyen Georges Poitou, 91400, Orsay, France.
[b]Université de Tunis El Manar, Faculté des Sciences, Laboratoire de Chimie Analytique et Electrochimie (LR99ES15), Campus universitaire de Tunis El Manar, 2092 Tunis El–Manar, Tunisia
[c]Synchrotron-SOLEIL, Saint-Aubin, BP48, F91192, Gif sur Yvette Cedex, France

SI.1. Material and methods

SI.1.1. Synthesis of the naphthoquinone derivative

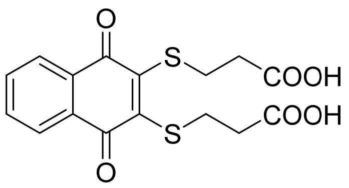

^{1}H NMR (d6-DMSO) δ 2.65–2.69 (t, 2H), 3.11–3.12 (t, 2H), 6.77 (s, 1H), 7.81–7.86 (m, 2H), 7.95–7.97 (m, 2H); 13C NMR (d6-DMSO) δ 24.76, 31.95, 126.03, 126.34, 127.28, 131.43, 131.68, 133.65, 134.74, 153.40, 172.48, 180.95, 181.83;

SI.1.2. Preparation of MoS_2 nanosheets

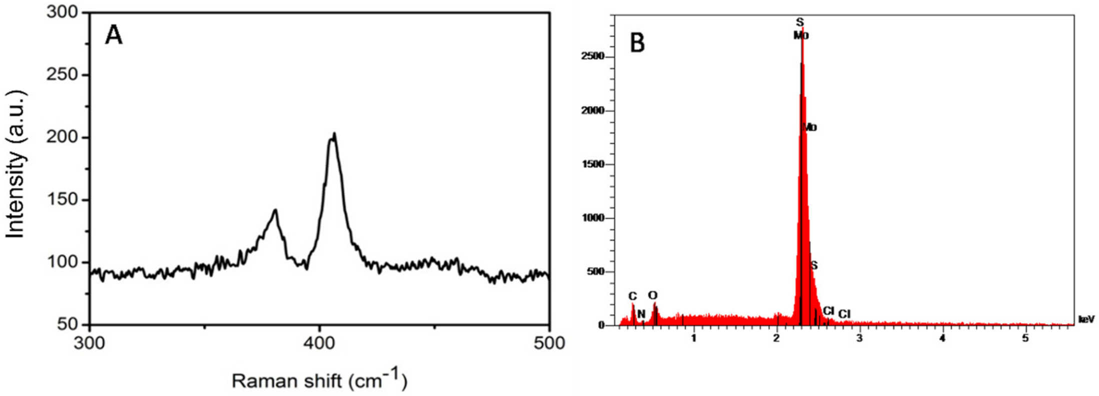

Figure S1. (**A**) Raman Spectra of MoS2 nanosheets and (**B**) EDX analysis of the modified SPCE with MoS_2 nanosheets

SI.1.3. Electrodeposition of MoS_2 on SPCE

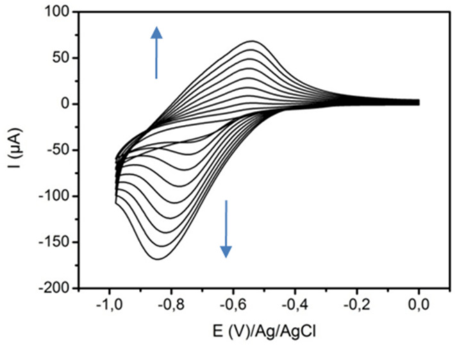

Figure S2. Electrodeposition of $MoS_2$by cycling the potential between -0.1V and -0.95V, for 10 cycles at scan rate 100 mV.s^{-1} in 0.5 M $LiClO_4$.

SI.1.4. Electropolymerization of PPyNPs on SPCE

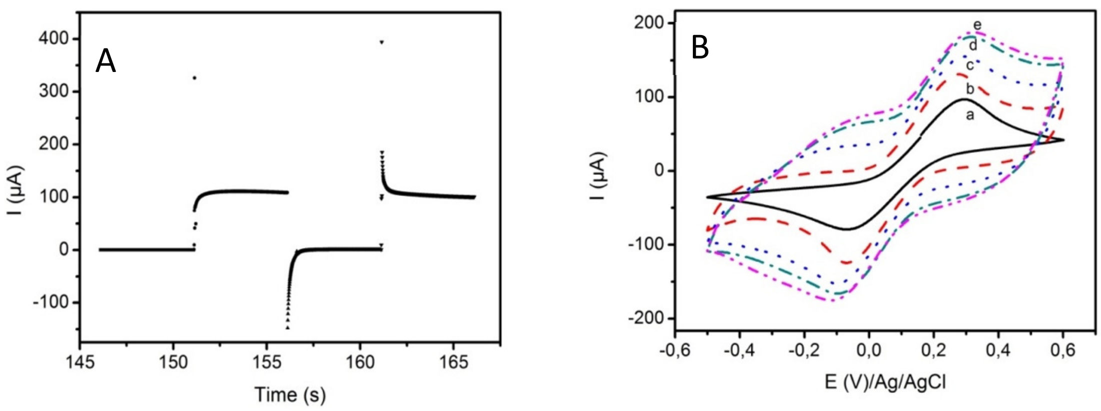

Figure S3. Electropolymerization of PPy by 2 pulses with Eapp = 0.8 V during 5s in 0.5M $LiClO_4$**(A)** CVs recorded in 5 mM $[Fe(CN)_6]^{3/4-}$ at v =100 mV.s^{-1} before and after PPy elecropolymerization **(B.a)** SPCE **(B.b)** 1 pulse **(B.c)** 2 pulses **(B.d)** 3 pluses and **(B.e)** 4 pulses

SI.1.5. Electrodeposition of EDA and Cystamine

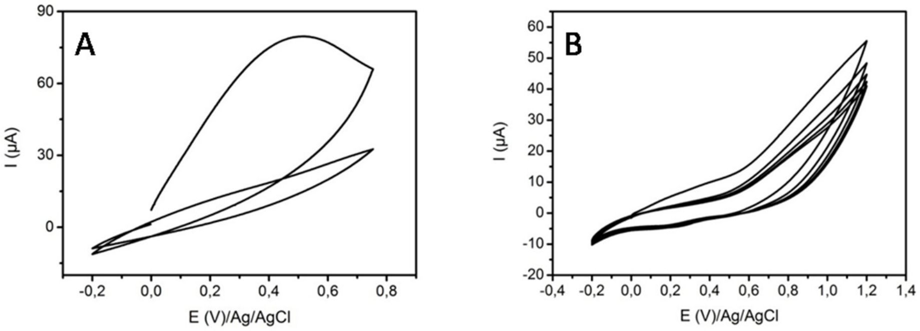

Figure S4. (**A**)CVs of Electrodeposition of EDA by cycling the potential between -0.2V and 0.8V, for 2cycles at scan rate 200 $mV.s^{-1}$, in 0.5 M $LiClO_4$, (**B**) Electrodeposition of Cystamine by cycling the potential between -0.2V and 1.2V, for 4 cycles at scan rate 100 mV/s, in 0.5 M $LiClO_4$

SI.2 Results and discussion

SI.2.1 Properties of MoS_2 and PPy

SI.2.1.1 Structural and morphological studies

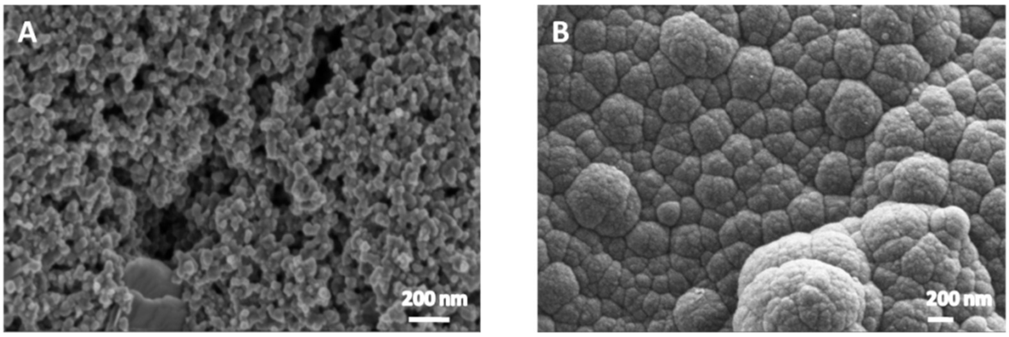

Figure S5. SEM images of (**A**) SPCE (**B**) SPCE/PPy Polypyrrole deposits using cyclic voltammetry

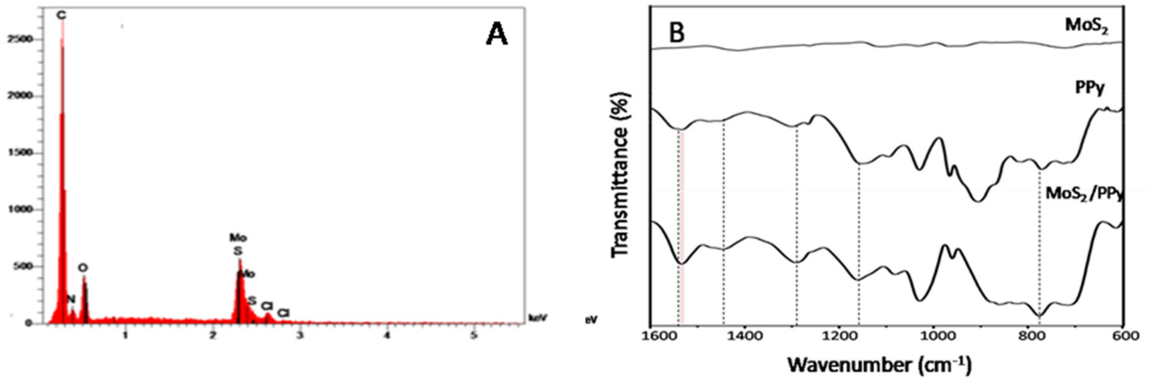

Figure S6. (A) EDX analysis of the modified SPCE with MoS_2/PPy and **(B)** FT-IR MoS_2 nanosheets, PPy and MoS_2/NPsPPy

SI.2.1.2. Electrochemical characterization of the nanocomposite

Table S1. Fitting parameters of EIS data

Equivalent circuit model	Modified electrode	R_s /Ω	R_{ct} /Ω	Capacitance µF	CPE1 µMho	n	CPE2 µMho	n	W µMho	χ^2
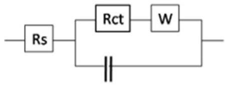	SPCE	321.01	849.7	0.976	-	-	-	-	669	0.1
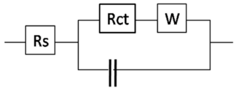	SPCE/MoS_2	316.7	411.61	1.19	-	-	-	-	1410	0.029
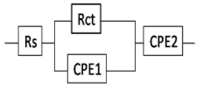	SPCE/PPy	303.34	197.14	-	288	0.54	860	0.95	-	0.001
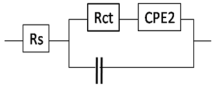	SPCE/MoS_2/PPy	306.55		22.7	-	-	2190	0.634	-	0.01

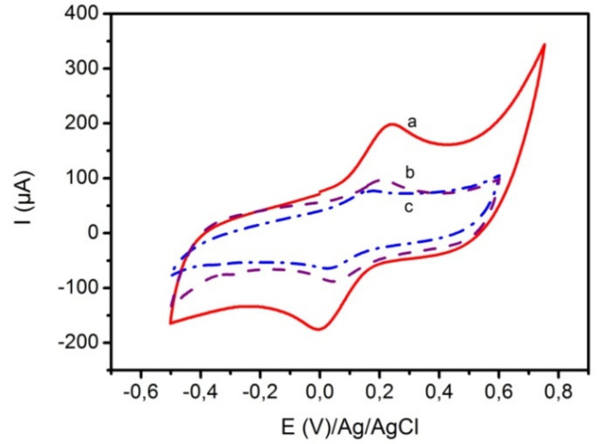

Figure S7. CVs recorded in presence of 5 mM $[Fe(CN)_6]^{3/4-}$ (**a**) SPCE/MoS_2/PPy ; (**b**) SPCE/MoS_2/PPy/EDA ;(**c**) SPCE/MoS_2/PPy/EDA/NQ ;

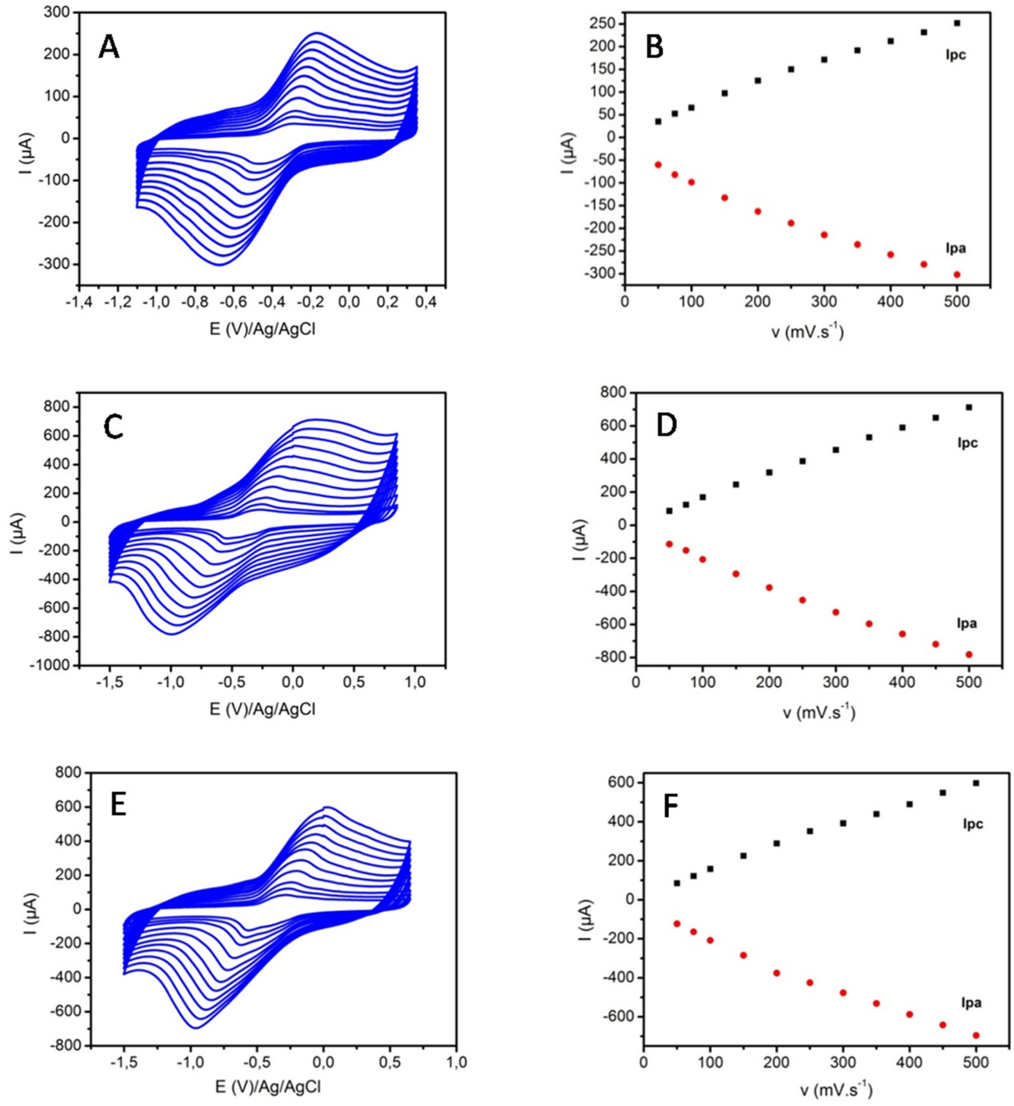

Figure S8. CVs of the redox signal of NQ in various modified SPCE in PBS pH= 7.4 with scan rate from 50 to 500 mV.s $^{-1}$ and Linear relationship of the redox peak currents versus scan rate **(A, B)** MoS_2/Cyst/NQ, **(C, D)** PPy/EDA/NQ, **(E, F)** MoS_2/PPy/EDA/NQ.

SI.2.2. Electrochemical characterization of the biolayers

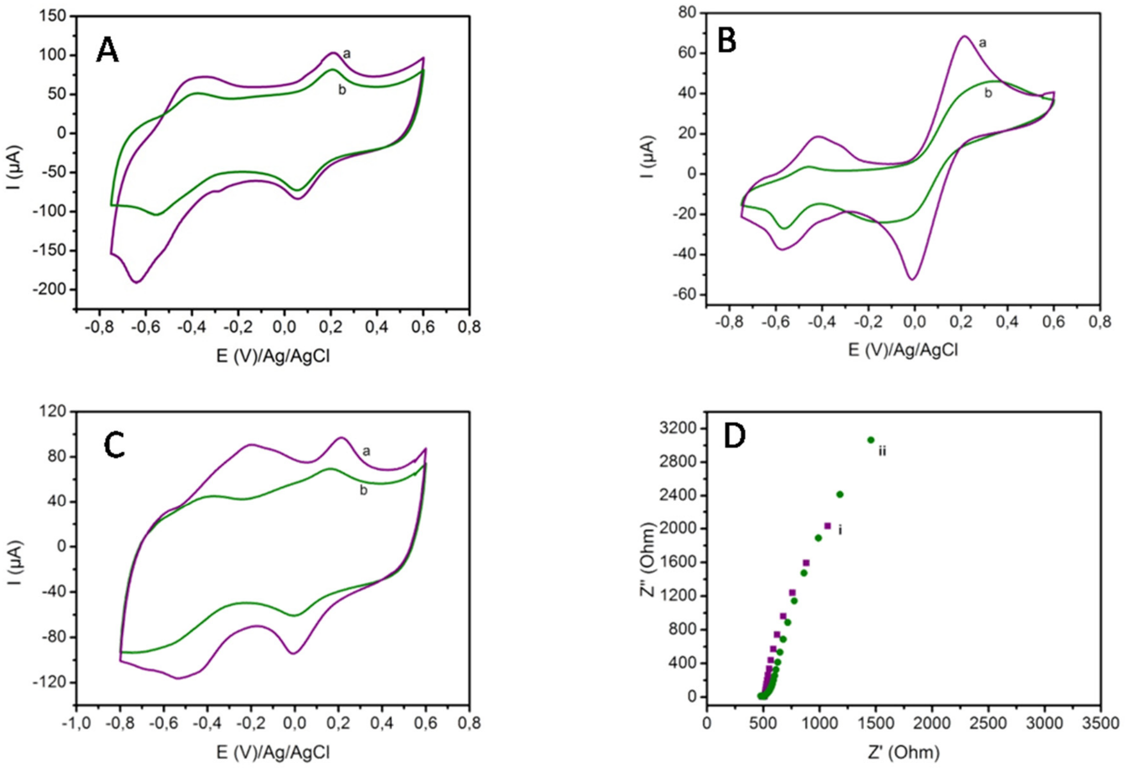

Figure S9. CVs recorded in presence of 5 mM $[Fe(CN)_6]^{3/4-}$ before and after aptamer immoabilization (**A.a**) SPCE/MoS2/PPy/EDA/NQ; (**A.b**) SPCE/MoS_2/PPy/EDA/NQ/APT; (**B.a**) SPCE/MoS_2/Cyst/NQ, (**B.b**) SPCE/MoS_2/Cyst/NQ /APT; (**C.a**) SPCE/PPy/EDA/NQ; (**C.b**) SPCE/PPy/EDA/NQ/APT; EIS (Nyquist plots) in presence of 5 mM $[Fe(CN)_6]^{3/4-}$ obtained before (**D**) (i) and after (**D**) (ii) aptamer immobilization (Eapp= 0.2V) Frequency range: 100 KHz to 0.1 Hz.

SI.2.3. Comparison of sensing platforms based on MoS_2

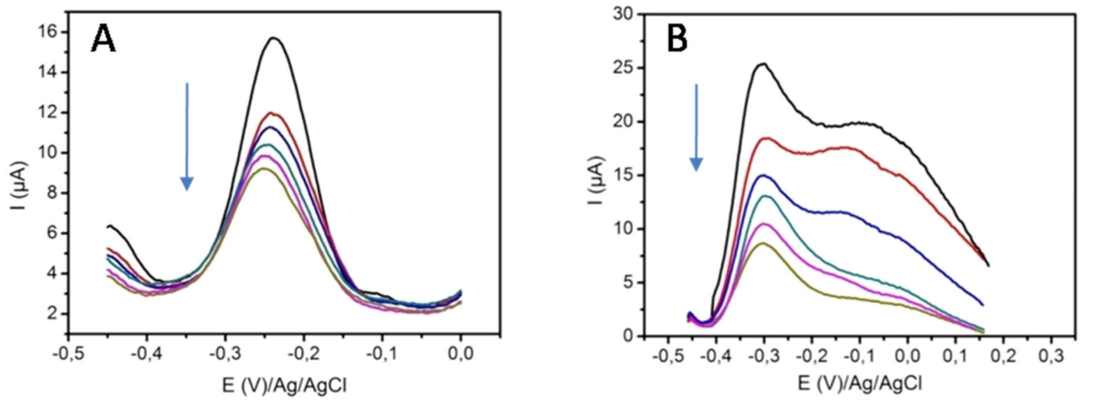

Figure S10. SWV of the oxidation peak after incubation at various ampicillin concentrations from 50 to 250 pg/L in PBS for **(A)** MoS_2 based electrode **(B)** PPy based electrode

SI.2.4. Detection in waste water

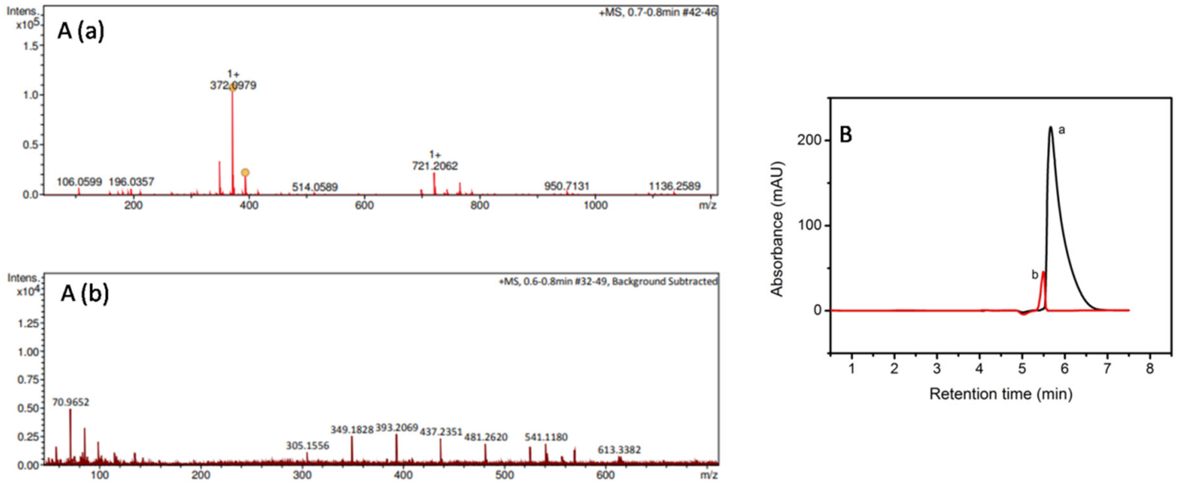

Figure S11. Mass Spectra of (a) water doped with AMP and (b) river water (B) High performance liquid chromatography (HPLC) analysis of (a) Ampicillin solution and (b) waste water sample
